# Patients Response to Interventional Care for Chronic Pain Study (PRICS): A Cross-Sectional Survey of Community-Based Pain Clinics in Ontario, Canada

**DOI:** 10.7759/cureus.37440

**Published:** 2023-04-11

**Authors:** Roman D Jovey, Jeffrey Balon, Joanne Mabee, Julie Yake, Candace Currer, Geeta Vadgama, Jane Jomy, Ker-Yung Hong, Mansi Patel, Jason W Busse

**Affiliations:** 1 Department of Pain Management, NeuPath Health Inc, Mississauga, CAN; 2 Department of Pain Management, NeuPath Health Inc, Ottawa, CAN; 3 Department of Clinical Research, NeuPath Health Inc, Toronto, CAN; 4 Department of Information Systems, NeuPath Health Inc, London, CAN; 5 Regional Director, NeuPath Health Inc, Toronto, CAN; 6 Regional Director, NeuPath Health Inc, Mississauga, CAN; 7 Department of Health Research Methods, Temerty Faculty of Medicine, University of Toronto, Toronto, CAN; 8 Department of Kinesiology, Faculty of Science, McMaster University, Hamilton, CAN; 9 Department of Health Research Methods, Evidence and Impact, McMaster University, Hamilton, CAN; 10 Department of Anesthesia, McMaster University, Hamilton, CAN

**Keywords:** thematic analysis, patient response, nerve blocks, outcomes, injections, community clinics, treatment, chronic non-cancer pain

## Abstract

Background: Non-image guided injection treatments (“nerve blocks”) are commonly provided in community pain clinics in Ontario for chronic non-cancer pain (CNCP) but remain controversial.

Aim: We explored patients’ perspectives of nerve blocks for CNCP.

Methods: We administered a 33-item cross-sectional survey to patients living with CNCP pain attending four community-based pain clinics in Ontario, Canada. The survey captured demographic information and asked about patient experiences with nerve blocks.

Results: Among 616 patients that were approached, 562 (91%) provided a completed survey. The mean age of respondents was 53 (SD 12), 71% were female, and the majority (57%) reported living with CNCP for more than a decade. Fifty-eight percent had been receiving nerve blocks for their pain for >3 years, 51% on a weekly frequency. Since receiving nerve blocks, patients self-reported a median improvement in pain intensity of 2.5 points (95% CI -2.5 to -3.0) on an 11-point numeric rating scale and 66% reported stopping or reducing prescription medications, including opioids. The majority who were not retired (62%) were receiving disability benefits and were unable to work in any capacity. When asked what impact cessation of nerve blocks would have, most employed patients (52%) reported they would be unable to work, and the majority indicated their ability to function across multiple domains would decrease.

Conclusion: Our respondents who received nerve blocks for CNCP attribute important pain relief and functional improvement to this intervention. Randomized trials and clinical practice guidelines are urgently needed to optimize the evidence-based use of nerve blocks for CNCP.

## Introduction

Chronic non-cancer pain (CNCP) has a high burden of illness, affecting approximately 20% of the population globally [1-3]. Chronic pain is associated with interference with physical functioning, daily activities, mental health, and social and family functioning and is a significant cause of lost productivity and disability in the workplace. The total direct (health care) and indirect (lost productivity) burden in Canada is estimated at $38-40 billion CAD annually. The direct healthcare costs are an estimated $15-17 billion CAD annually, which is approximately 10% of the total Canadian healthcare budget [3].

Medical management of chronic pain in Canada has generally focused on pharmacotherapy [4]. There are, however, increasing concerns regarding medication-related harms, particularly with opioids, as well as increasing awareness of the modest benefits of pharmacologic therapy [5-10]. Interventional procedures, such as non-image-guided nerve blocks, paravertebral blocks, joint, trigger point and scar injections, utilizing local anesthetic agents with or without corticosteroids, have been increasingly used in Ontario for CNCP. Ontario’s tax-funded public health care plan (OHIP) reimbursed an estimated $420 million for nerve blocks in community and hospital clinics from 2011 to 2020 [11]. However, these interventions have come under criticism due to conflicting evidence for effectiveness and concerns that some physicians providing these services may prioritize revenue over optimal patient care [12-14]. Moreover, a 2021 draft standard from the College of Physicians and Surgeons of Ontario has proposed that, except for superficial facial nerve blocks, physicians administering nerve blocks for adult chronic pain must use image guidance [15]. We conducted a survey to assess patients’ perspectives regarding nerve blocks for CNCP.

## Materials and methods

With the assistance of content experts, we developed a 33-item, English-language questionnaire to capture the experiences and impressions of patients with CNCP who receive nerve blocks (Appendix 1). Numeric rating scales for outcomes and questions from the Pain Disability Index were embedded in this survey. In January 2017, we conducted a pilot survey with 31 patients to evaluate if the questionnaire adequately measured views towards, and experiences with, non-image-guided nerve blocks for CNCP. The pre-test participants were also asked to comment on the clarity and comprehensiveness of the questionnaire and the time required for completion. Feedback resulted in minor wording changes in the survey to improve comprehension and the addition of questions regarding medication side effects, and the duration of pain relief after nerve blocks. The final questionnaire provided response options as checkboxes, as a previous report has shown that closed-ended questions result in fewer incomplete questionnaires compared to open-ended questions [16]. We also included an open-ended question to allow respondents to provide additional information if they wished to do so.

Questionnaire administration

Patients were recruited from four Canadian, privately owned, community-based chronic pain clinics in Ontario, NeuPath Centres for Pain and Spine. Approximately 80% of active patients attending these clinics receive therapeutic nerve blocks which pragmatically refers to a combination of specific peripheral nerve blocks, paravertebral blocks, trigger point, scar and joint injections, using local anesthetics, such as lidocaine, bupivacaine, ropivacaine, and mepivacaine, occasionally combined with steroids. About 20% of patients attending this group of clinics discontinued blocks within the first month due to lack of efficacy and 56% continued to receive blocks for at least one year.

From February 2019 to March 2020, all eligible patients attending each of the four clinics were notified, by mail or in-person, by the research team, and offered the opportunity to participate in our survey. Patients attending the clinic frequently (every two weeks or less) were approached in-person by administrative staff, and those attending less frequently were sent a letter by mail. The survey was administered at one clinic at a time for 4-6 weeks. Patients eligible to participate were (1) 18 years of age or older, (2) living with CNCP for >12 months, (3) currently receiving interventional nerve blocks, with a minimum of six treatments in the past six months, (4) fluent in English, and (5) provided written informed consent.

Patients were not excluded because of physical or mental comorbidities. Patients who agreed to participate were given a study subject number and advised that their answers to the survey would be confidential and their pain care would not be affected by their answers to the survey. Only the lead researchers (RJ and JB) and members of the research team had access to survey data and patients’ treating physicians were unaware of who participated in the study. We originally planned to survey 1000 patients but discontinued recruitment prematurely due to COVID-19 restrictions. Our study methodology and survey were approved by the Advarra Institutional Review Board (https://www.advarra.com; project CR00142112).

Data management and storage

After eligible patients provided informed consent to participate, they were asked to complete the questionnaire during a regular treatment visit. Data was collected on an electronic tablet, using the Ocean software platform (https://www.cognisantmd.com). After providing instruction on the use of the tablet, research staff entered an anonymous participant code for each individual patient, who then completed the survey independently. Patients had an opportunity to review and edit responses prior to submitting their data. All survey data was automatically entered into a validated, password-protected, secure database that was only accessible to members of the study team.

Statistical analysis

We reported categorical data as proportions and continuous data as means and standard deviations (SDs) if normally distributed and as medians and interquartile ranges (IQRs) if not. We assessed the normality of continuous data with the Kolmogorov-Smirnov (K-S) test. As pain scores and quality of life scores were not normally distributed (K-S test p<0.001 for all values), we used a Wilcoxon matched-pair rank test to explore differences in median values within respondents and estimated the 95% confidence interval (95% CI) associated with the median change with the Hodges-Lehmann test. We performed all analyses using IBM SPSS 26.0 statistical software (Armonk, NY: IBM Corp). All comparisons were two-tailed and we used a p-value <0.05 for statistical significance. Two of us (K-YH, MP) reviewed written comments independently and in duplicate to establish common themes and representative quotes. Any discrepancies were resolved by discussion.

## Results

Among 616 subjects that were eligible for inclusion, 54 declined for a participation rate of 91% (562 of 616). The mean age of respondents was 53 (SD= 12), 71% were female, and approximately half were married. The majority (57%) had lived with CNCP for more than a decade and had been attending their current pain clinic for more than three years. Further, 31% of respondents had attended their current clinic for more than five years. The majority who were not retired (62%) were receiving disability benefits and were unable to work in any capacity (Table 1).

**Table 1:** Demographic characteristics of participants

	No. (%) of respondents
Gender (n=568)	
Male	166 (29%)
Female	402 (71%)
Age, mean (SD), (n=569)	53 (12)
Marital status (n=572)	
married	274 (48%)
single	179 (32%)
other	115 (20%)
No. of children (n=572)	
0	147 (26%)
1	89 (16%)
2	198 (35%)
3	80 (14%)
>4	58 (11%)
Employment status (n=572)	
Working full-time	122 (21%)
Working part-time	50 (9%)
Not working, on disability	283 (50%)
Not working, retired	117 (21%)
Annual gross income (n=572)	
$0 to 19,000	246 (43%)
$20,000 to 39,000	155 (27%)
$40,000 to 59,000	74 (13%)
$60,000 to 79,000	48 (8%)
$80,000 to 99,000	25 (4%)
≥$100,000	24 (4%)
Duration of chronic pain (n=571)	
<1 year	1 (0.2%)
1 to <2 years	10 (2%)
2 to <3 years	18 (3%)
3 to <5 years	61 (11%)
5 to <7 years	74 (13%)
7 to 10 years	83 (15%)
>10 years	324 (57%)
Length of time attending the current pain clinic (n=565)	
0 to <12 months	56 (10%)
1 to <2 years	128 (23%)
2 to <3 years	99 (18%)
3 to 5 years	108 (19%)
>5 years	174 (31%)

Table 2 presents the areas of life most affected by chronic pain reported by respondents. Most respondents indicated that every aspect of their lives was negatively impacted by their pain, including the ability to exercise (92%), work around the house (91%), sleep (91%), work outside the home (89%), and mood (87%).

**Table 2:** Areas of life most affected by chronic pain

Areas of life most affected by chronic pain	No. (%) of respondents
regular work outside the home	506 (89%)
work around the house	522 (91%)
relationships with family	418 (73%)
relationships with others	417 (73%)
ability to exercise or participate in sports	524 (92%)
ability to participate in hobbies or recreation	493 (86%)
finances	337 (59%)
mood	497 (87%)
sleep	519 (91%)

Prior to attending their current pain clinic, most respondents had received plain films (85%) and advanced imaging (e.g. MRI 85%, CT scan 67%). Almost all (95%) had seen a family physician, and many had previously attended medical specialists and surgeons regarding their pain. Most had received physiotherapy, massage, and chiropractic care. Almost all (99%) had been prescribed pharmacological treatments, and 28% had prior experience with nerve blocks. Self-reported side effects of the medication were common, with the majority endorsing dry mouth (69%), constipation (59%), impaired sleep (51%), and nausea (50%). Perceived helpfulness of prior referrals and treatments was highly variable, with the greatest endorsement for nerve blocks (median of 8 on a 10-point scale, IQR= 6-9). Sixty-six percent of patients self-reported stopping or reducing non-prescription and prescription medications since starting nerve blocks, including a reduction in opioid use (Tables 3-6).

**Table 3:** Treatment sought prior to management at the current pain clinic ^1.^ Of those who attended a prior pain clinic, 76% (121 of 160) had attended a single clinic, 18% (n=28) had attended two clinics, and 7% (n=11) had attended three or more clinics. ^2. ^The scale ranged from 0 (not at all helpful) to 10 (extremely helpful).

Another pain clinic (n=572)	No. (%) of respondents	
Yes	156 (27%)^1^	
No	416 (73%)	
Conventional therapies (n=572)	pursued, no. (%)	perceived helpfulness (0-10)^2^, median (IQR)
nerve blocks	160 (28%)	8 (6 to 9)
epidurals	60 (11%)	5 (2 to 8)
medication	569 (99%)	6 (4 to 8)
psychotherapy	54 (9%)	5 (3 to 7)
self-management	91 (16%)	5 (2 to 7)
Alternative therapies (n=572)	pursued, no. (%)	perceived helpfulness (0-10)^2^, median (IQR)
natural supplements	197 (34%)	2 (1 to 4)
physiotherapy	396 (69%)	3 (2 to 5)
chiropractic	293 (51%)	3 (2 to 5)
acupuncture	263 (46%)	3 (1 to 5)
massage	350 (61%)	4 (2 to 6)
meditation/hypnosis	162 (28%)	4 (2 to 5)
Previous medical specialists seen prior to current clinic (n=572)	pursued, no. (%)	perceived helpfulness (0-10)^2^, median (IQR)
family doctor	543 (95%)	4 (3 to 6)
rheumatologist	189 (33%)	4 (2 to 6)
physiatrist	171 (30%)	3 (2 to 6)
neurologist	222 (39%)	3 (2 to 6)
orthopedic surgeon	190 (33%)	5 (2 to 7)
neurosurgeon	92 (16%)	3 (1 to 6)

**Table 4:** Investigations sought prior to management at the current pain clinic

Investigations prior to current clinic (n=572)	no. (%) of respondents endorsing investigations	no. of investigations done, median (IQR)
CT scan	383 (67%)	2 (1 to 3)
MRI	491 (86%)	2 (1 to 3)
Ultrasound	290 (51%)	2 (1 to 4)
X-ray	487 (85%)	3 (2 to 6)
Bone scan	227 (40%)	1 (1 to 2)

**Table 5:** Previous and current medications prescribed for respondents, and impact of blocks on medication use ^1. ^Of 571 respondents, 194 (34%) had not stopped or reduced any medications since starting nerve blocks, and 377 (66%) had. ^2. ^The proportion of patients that reported stopping or reducing medication use was often inconsistent with the differences between previously and currently prescribed, which reflects a limitation of self-reported cross-sectional data. NSAIDs: non-steroidal anti-inflammatory drugs

Medication	Previously prescribed (n=572)	Currently prescribed (n=572)	Stopped or reduced use after starting blocks (n=377)^1,2^
topical rubs	429 (75%)	236 (41%)	100 (27%)
acetaminophen	480 (84%)	187 (33%)	149 (40%)
NSAIDs	329 (58%)	103 (18%)	109 (29%)
codeine	374 (65%)	96 (17%)	126 (33%)
oxycodone	318 (56%)	166 (29%)	153 (41%)
hydromorphone	195 (34%)	81 (14%)	97 (26%)
morphine	151 (26%)	51 (9%)	64 (17%)
tramadol	179 (31%)	53 (9%)	63 (17%)
tapentadol	20 (4%)	16 (3%)	13 (3%)
fentanyl patch	91 (16%)	27 (5%)	53 (14%)
buprenorphine	43 (8%)	29 (5%)	14 (4%)
tricyclics	171 (30%)	48 (8%)	34 (9%)
gabapentin	310 (54%)	188 (33%)	83 (22%)
duloxetine	218 (38%)	114 (20%)	40 (11%)
topiramate	81 (14%)	41 (7%)	18 (5%)
nabilone	119 (21%)	90 (16%)	30 (8%)

**Table 6:** Previous experience with medication side effects (n=572)

Adverse events associated with medication use	Proportion of respondents who previously experienced each side effect due to medication use, no. (%)
nausea	285 (50%)
headaches	238 (42%)
constipation	336 (59%)
low blood pressure	57 (10%)
irregular heart rhythms	51 (9%)
muscle spasms	176 (31%)
dry mouth	392 (69%)
impotence	60 (11%)
drowsiness	274 (48%)
sleep difficulty	293 (51%)
confusion	133 (23%)
rash	60 (11%)
increasing pain	130 (23%)

Table 7 presents patients’ experiences receiving nerve blocks at their current pain clinic. The majority of respondents (58%) had been receiving nerve blocks for chronic pain for greater than three years, at intervals ranging from weekly (51%) to longer than every three weeks (20%). Most respondents endorsed experiencing pain relief that lasted less than seven days.

**Table 7:** Experiences receiving nerve blocks at the current pain clinic

Duration of receiving nerve blocks (n=564)	no. (%) of respondents
< 1 year	68 (12%)
1-2 years	167 (30%)
3-4 years	133 (23%)
> 4 years	196 (35%)
Frequency of receiving nerve blocks (n=558)	
at least weekly	282 (51%)
every 2 weeks	164 (29%)
every 3 weeks or more	112 (20%)
Onset of pain relief after nerve blocks (n=564)	
immediately	283 (50%)
within 1 hour	149 (26%)
within 4 hours or more	132 (24%)
Length of pain relief after nerve blocks (n=558)	
< 2 days	63 (11%)
3-6 days	287 (51%)
7-10 days	118 (21%)
> 2 weeks	90 (16%)

Table 8 presents patients’ pain before and after nerve blocks. When asked to recall their average pain severity before and after nerve blocks in general, patients reported a median improvement on an 11-point numeric rating scale (NRS) of 2.5 points (95% CI= -2.5 to -3.0).

**Table 8:** Pain before and after nerve blocks* ^1. ^The Wilcoxon matched-pair rank sign test was used to estimate the difference in median scores before and after nerve blocks. * The overall helpfulness of nerve blocks to reduce pain, measured on a 0 to 10 scale with higher scores meaning greater satisfaction, was a median of 8 (IQR 7 to 9) (n=566).

	Before (0-10), median (IQR)	After (0-10), median (IQR)	Median of the difference (95%CI)^1^	p-value for difference
Worst pain (n=564)	9 (8 to 10)	5 (3 to 7)	-4 (-4.0 to -3.5)	p<0.001
Least pain (n=561)	6 (5 to 8)	3 (2 to 6)	-2.5 (-3.0 to -2.5)	p<0.001
Average pain (n=563)	7 (6 to 8)	5 (3 to 6)	-2.5 (-3.0 to -2.5)	p<0.001

An improvement of 2-point is the minimally important difference for the 11-point NRS for pain [17]. Using this threshold, 74% of respondents (416 of 562) reported an improvement in average pain that met or exceeded the minimally important difference. Table 9 presents patients’ quality of life before and after nerve blocks. Patients recalled low levels of functioning across a range of domains (e.g., occupation, self-care) prior to beginning nerve block treatments, ranging from a median of 1 to 4 on an 11-point NRS. They perceived important improvements in every domain because of their nerve block treatments, with median increases on the same scale ranging from 4 points (family responsibilities) to 2 points (sexual behaviour). Table 10 presents the side effects patients experienced after receiving nerve blocks. Only 7% of respondents reported any adverse events associated with nerve blocks.

**Table 9:** Quality of life before and after nerve blocks* ^1. ^The Wilcoxon matched-pair rank sign test was used to estimate the difference in median scores before and after nerve blocks. * Overall helpfulness of nerve blocks for improving function and quality of life, measured on a 0 to 10 scale with higher scores meaning greater helpfulness, was a median of 7 (IQR 6 to 8) (n=552).

	Before nerve blocks (0-10), median (IQR)	After nerve blocks (0-10), median (IQR)	Median of the difference (95%CI)^1^	p-value for difference
Family/home responsibilities (n=566)	2 (1 to 4)	7 (5 to 8)	4 (4 to 4)	p<0.001
Recreation (n=562)	2 (1 to 3)	6 (4 to 7)	3.5 (3.5 to 4.0)	p<0.001
Social activity (n=562)	2 (1 to 4)	6 (5 to 8)	3.5 (3.5 to 4.0)	p<0.001
Occupation (n=539)	1 (0 to 3)	6 (3 to 7)	3.0 (2.5 to 3.0)	p<0.001
Sexual behaviour (n=537)	2 (1 to 4)	5 (3 to 7)	2.0 (2.0 to 2.5)	p<0.001
Self-care (n=563)	4 (2 to 6)	7 (6 to 9)	3.0 (3.0 to 3.5)	p<0.001
Life-support activities (n=563)	4 (2 to 6)	7 (6 to 9)	3.0 (2.5 to 3.0)	p<0.001

**Table 10:** Side effects of blocks ^1. ^Of the 42 patients that experienced significant side effects after nerve blocks, 7% (three of 42) considered stopping nerve blocks as a result.

Experienced significant side effects after nerve blocks (n=572)	No. (%) of respondents
yes^1^	42 (7%)
no	530 (93%)
Side effects (n=572)	
nausea	10 (2%)
headaches	11 (2%)
constipation	13 (2%)
low blood pressure	5 (1%)
irregular heart rhythms	6 (1%)
muscle spasms	9 (2%)
dry mouth	23 (4%)
impotence	6 (1%)
drowsiness	16 (3%)
insomnia	7 (1%)
confusion	6 (1%)
rash	4 (1%)
bruising	14 (2%)
swelling	12 (2%)
weakness in a limb (arm or leg)	19 (3%)
passing out	2 (1%)
increasing pain	9 (2%)

When asked about the impact if the provincial government defunded nerve blocks for CNCP, most employed patients (52%) reported they would be unable to work in any capacity, the large majority indicated their ability to function across multiple domains would decrease, and most anticipated increased visits to their family physician and emergency department and greater use of prescription medication (Table 11).

**Table 11:** Perceived impact of defunding nerve blocks* * for the question of perceived helpfulness of nerve blocks for returning to, or maintaining employment among 157 respondents who were working, on a 0-10 scale, the median was: 8

Impact of defunding nerve blocks on work, no. (%), (n=161)	
could not work full time	71 (44%)
could not work at all	84 (52%)
no effect	6 (4%)
Impact of defunding nerve blocks, no. (%), (n=572)	
less ability to provide self-care	460 (80%)
less ability to perform home chores	538 (94%)
less ability to be a good parent or spouse	426 (75%)
less ability to enjoy hobbies or recreational activities	534 (93%)
less ability to socialize	511 (89%)
worse mood	528 (92%)
worse sleep	536 (94%)
Anticipated reactions if nerve blocks defunded, no. (%), (n=572)**	
more family doctor visits	458 (80%)
more emergency department visits	306 (54%)
more prescription pain medication	496 (87%)
seek out surgery	189 (33%)

Written comments

Written comments regarding nerve blocks were provided by 378 respondents and these were grouped into six themes: (1) increased functioning, (2) improved mental health, (3) role in pain management, (4) improved quality of life, (5) decreased reliance on other pain relief methods, and (6) concerns regarding proposed defunding for nerve blocks for chronic pain (Table 12, Appendix 2). For example:

“Without nerve blocks, I wouldn’t be able to work, and I’d probably be dead by now.”

“Nerve blocks gave me my life back. I was able to plan things, accept invitations for Christmas, go to the movies, play golf, and enjoy with my friends. Without nerve blocks, I was just in pain. With nerve blocks, I got my life back and could feel joy again…”

“Nerve blocks saved me from a life of opioid addiction and gives me the ability to work and make an income to help support my family.”

## Discussion

Our survey of a group of adults with CNCP, attending four community-based chronic pain clinics in Ontario, Canada, found the large majority reported important and meaningful pain relief and improved quality of life that they attributed to the receipt of nerve blocks. The majority of respondents indicated that nerve blocks had allowed them to reduce or discontinue the use of non-prescription and prescription medications, including opioids. Reported adverse events associated with blocks were very rare. Patients reported that defunding of nerve blocks for chronic pain by the provincial government would have deleterious consequences. Despite these perceived improvements, most working-age patients were unemployed and receiving disability benefits.

Strengths and limitations

Strengths of our study include a high response rate (91%), providing assurances that our findings are likely representative of patients with CNCP attending the four community pain clinics in Ontario that we sampled, and the piloting of our survey prior to administration.

There are several limitations of this study. First, selection bias; our respondents, by inclusion criteria, were already responders to interventional treatments. Second, recall bias; our cross-sectional survey asked patients to remember their pain and functional status, and medication use, prior to starting nerve blocks and compared to their current status. As an example, respondents’ replies to medications they had reduced or stopped due to nerve blocks were inconsistent with the difference between previously and currently prescribed (Table 5). Third, financial conflicts of interest; this study was funded by a private pain clinic organization and the physician co-authors working in these clinics have a vested interest in the study results; however, all data and thematic analyses were conducted by an independent group at McMaster University. Fourth, our 33-item questionnaire has not been validated. Fifth, participants may have censored their answers to appear as “good patients” (i.e., social desirability bias). Sixth, the observational nature of our study design cannot establish causation between the receipt of nerve blocks and reported outcomes.

Relevant literature

Our respondents indicated a large net benefit with nerve blocks for CNCP: important reduction in pain and improved function and quality of life with few adverse events. Another cross-sectional survey of Ontario community-based pain clinics found similar results to ours [13]. However, self-perceived improvement in pain and function may not translate into employment, and most respondents in our sample were receiving disability benefits. At the same time, return to work may be influenced by other factors, such as time out of the workforce, bureaucratic barriers, lack of funding support for remedial skills acquisition, and concerns over losing wage replacement benefits [18,19].

All outpatient interventional pain clinics in Ontario are obligated to report serious adverse events that occur within 10 days of receiving treatment to the College of Physicians and Surgeons of Ontario Out of Hospital Premises Inspection Program (OHPIP). These include any patients being transferred to the ER after receiving a nerve block and all deaths from any cause within 10 days of treatment. Among the four clinics that administered our survey to their patients, there have never been more than 10 reportable events per year - the majority being vasovagal events with spontaneous recovery after a short period of observation. There were no deaths attributed to treatment.

Our respondents self-reported reduced use of prescription medication, including opioids, after starting nerve block therapy. In contrast, a retrospective analysis of 47,723 patients in Ontario, Canada, who received nerve blocks for CNCP between 2013 and 2018 found no change in mean opioid dose between the year before and the year after starting nerve block therapy. This study did not rely on self-report but accessed the Narcotics Monitoring System which tracks all opioids dispensed in the province of Ontario. There were, however, within patient differences; specifically, 43% increased their use of opioids, 17% had no change, and 40% reduced their prescribed opioids one year after starting nerve block therapy [20].

In 2020, the National Institute for Health and Care Excellence (NICE) recommended against spinal injections for managing chronic low back pain due to the lack of supporting evidence [21]. Also in 2020, the American Society of Interventional Pain Physicians (ASIPP) released their updated guideline reaffirming recommendations in favour of radiofrequency ablation, nerve blocks and facet joint injections for chronic low back pain [22]. One challenge with interpreting the evidence of therapies for CNCP, including nerve blocks, is the role of non-specific effects.

Consider a 2013 survey of 260 patients with CNCP attending a tertiary multidisciplinary pain clinic in Ontario. The majority (88%) were receiving long-term opioid therapy and no patients were receiving nerve blocks or other interventional procedures; 74% reported >40% pain relief and 68% reported >40% functional improvement. Consistent with our findings, most of these patients (68%) were disabled from working and receiving wage replacement benefits [23]. A systematic review of 96 randomized trials of opioid therapy for CNCP was able to account for non-specific effects. This study found that 61% of patients allocated to opioids reported important pain relief, however, so did 49% of those randomized to receive a placebo [6]. The non-specific effects of invasive procedures for CNCP are likely to be larger than pharmacotherapy [24,25].

## Conclusions

Our survey found that people living with CNCP that pursue and choose to continue with, nerve blocks reported important and meaningful benefits in pain and function as well as reduced medication use (including opioids) with few adverse effects. While observational studies provide insights into the experiences of patients, they cannot establish causality. Rigorously conducted controlled trials of nerve blocks for CNCP are required to inform effectiveness and evidence-based guidelines for interventional procedures and chronic pain, informed by both current best evidence and patients’ values and preferences, are urgently needed.

## Appendices

Appendix 1

Patient Response to Interventional Care Survey (PRICS)

Date:

Dear CPM Patient,

Please help us to improve our pain management services by answering a few questions about the effect that nerve blocks have had on your pain and your quality of life.

All the information that you provide below will be entered only as anonymous statistical information in a database. No one will know the names of any individual patients completing the survey.

1. Participant code  (Clinic#-patient#): _________________________________ 

2. Demographics:

a. Year of Birth: (drop down box) _____     Gender:  Male ____  Female___                                                                                                                                                     ( yyyy)                                                                                 

b. Marital status (drop down box): Married___ Single___ Other___

c. Children (drop down box): 0 ___ 1___ 2___ 3___ 4___ 5___ >5____

d. Are you currently employed?

• Yes, full-time

• Yes, full-time

• No, on disability

• No, retired

e. Annual income bracket if you are currently working (drop-down box):

 • $0-20,000

 • $20-40,000

 • $40-60,000

 • $60-80,000

 • $80-100,000

 • >$100,000

3. City where you live: _____________________________  

4. How long have you had chronic pain? 

       • < 1 year

       • 1-2 years

       • 2-3 years

       • 3-5 years

       • 5-7 years

       • 7-10 years

       • > 10 years                            

5. When did you first start coming to the CPM clinic? 

      • 0-6 months

      • 6-12months

      • 1-2 years

      • 2-3 years

      • 3-5 years

      • > 5 years

6. Were you ever treated at another pain clinic before coming to our clinic?                      (Yes/No)  

    If yes, how many other pain clinics? 

      • 1                                           • 6                                      • >10

      • 2                                          • 7

      • 3                                          • 8

      • 4                                          • 9

      • 5                                          • 10                      

7. If you were treated at another pain clinic before CPM, which treatments did you receive there, and indicate how helpful each was.

    (0=Not at all helpful to 10= extremely helpful):

      • Nerve blocks   (0    1    2    3    4    5    6    7    8    9    10)

      • Epidurals         (0    1    2    3    4    5    6    7    8    9    10)

      • Medications     (0    1    2    3    4    5    6    7    8    9    10)

      • Psychological counselling (0    1    2    3    4    5    6    7    8    9    10)

      • Chronic Pain Self Management (0    1    2    3    4    5    6    7    8    9    10)

      • Other treatments received:  __________________________________________                                                                                                           

8. Please check any other alternative treatments you tried before starting nerve blocks, and indicate how helpful each was.

       (0=Not at all helpful to 10= extremely helpful): 

      • Natural supplements                (0    1    2    3    4    5    6    7    8    9    10)

      • Physiotherapy                               (0    1    2    3    4    5    6    7    8    9    10)

      • Chiropractic                                    (0    1    2    3    4    5    6    7    8    9    10) 

      • Acupuncture                                  (0    1    2    3    4    5    6    7    8    9    10)

      • Massage                                             (0    1    2    3    4    5    6    7    8    9    10) 

      • Meditation/Hypnosis               (0    1    2    3    4    5    6    7    8    9    10)

      • Other: ______________________   (0    1    2    3    4    5    6    7    8    9    10)

   9. What other medical specialists have you seen before coming to CPM?  If you have seen more than one,     

      please indicate how many of each type of specialist you have seen 

      • Rheumatologist   ☐1      ☐2    ☐3    ☐4     ☐5     ☐>5

      • Rehabilitation medicine doctor   ☐1      ☐2    ☐3    ☐4     ☐5    ☐>5

      • Neurologist   ☐1      ☐2    ☐3    ☐4     ☐5     ☐>5

      • Orthopedic surgeon   ☐1      ☐2    ☐3    ☐4     ☐5    ☐>5

      • Neurosurgeon   ☐1     ☐2    ☐3    ☐4     ☐5     ☐>5 

      • Other_________________________ 

10. Please indicate how helpful each of the previous doctors that you saw for your chronic pain were

       (0=Not at all helpful to10= extremely helpful): 

      • Family doctor                                (0    1    2    3    4    5    6    7    8    9    10)

      • Neurologist                                    (0    1    2    3    4    5    6    7    8    9    10)

      • Neurosurgeon                               (0    1    2    3    4    5    6    7    8    9    10)

      • Rheumatologist                             (0    1    2    3    4    5    6    7    8    9    10) 

      • Physiatrist/Rehab Med                (0    1    2    3    4    5    6    7    8    9    10)

      • Orthopedic Surgeon                      (0    1    2    3    4    5    6    7    8    9    10) 

      • Other: _______________________ (0    1    2    3    4    5    6    7    8    9    10)

11. How often have you had each of the following investigations prior to coming to CPM? 

      • CT scan  ☐1      ☐2    ☐3    ☐4    ☐5     ☐>5

      • MRI          ☐1      ☐2    ☐3    ☐4    ☐5    ☐>5

      • Ultrasound  ☐1      ☐2    ☐3    ☐4     ☐5    ☐>5

      • X-rays      ☐1      ☐2    ☐3    ☐4    ☐5    ☐>5

      • Bone scan  ☐1      ☐2    ☐3    ☐4    ☐5     ☐>5

 12. Which medications have you tried before coming to CPM? (Check all that apply)

      • Topical rubs

      • Acetaminophen (Tylenol)

      • NSAIDs (Motrin, Naprosyn) 

      • Codeine (Tylenol #3, Codeine Contin) 

      • Oxycodone (Percocet, OxyContin, OxyNEO) 

      • Hydromorphone (Dilaudid, Jurnista, Hydromorph Contin) 

      • Morphine (MS Contin, MEslon, Kadian) 

      • Tramadol (Ultram, Tramacet, Tridural, Durela) 

      • Tapentadol (Nucynta)  

      • Fentanyl patch 

      • Buprenorphine (BuTrans patch)

      • Buprenorphine buccal film (Belbuca)

      • Tricyclics (Amitriptyline, Nortriptyline) 

      • Gabapentin (Neurontin), Pregabalin (Lyrica) 

      • Duloxetine (Cymbalta), Venlafaxine (Effexor)

      • Topiramate (Topamax) 

      • Nabilone (Cesamet) 

      • Other: _______

13.  What medications are you taking now?  (Check all that apply)

      • Topical rubs

      • Acetaminophen (Tylenol)

      • NSAIDs (Motrin, Naprosyn) 

      • Codeine (Tylenol #3, Codeine Contin) 

      • Oxycodone (Percocet, OxyContin, OxyNEO) 

      • Hydromorphone (Dilaudid, Jurnista, Hydromorph Contin) 

      • Morphine (MS Contin, MEslon, Kadian) 

      • Tramadol (Ultram, Tramacet, Tridural, Durela) 

      • Tapentadol (Nucynta)  

      • Fentanyl patch 

      • Buprenorphine (BuTrans patch) 

      • Buprenorphine buccal film (Belbuca)

      • Tricyclics (Amitriptyline, Nortriptyline) 

      • Gabapentin (Neurontin), Pregabalin (Lyrica) 

      • Duloxetine (Cymbalta), Venlafaxine (Effexor) 

      • Topiramate (Topamax) 

      • Nabilone (Cesamet) 

      • Other: _______

14. What significant medication side effects have you previously experienced? (Check all that apply)

      • Nausea

      • Headaches

      • Constipation 

      • Low Blood Pressure 

      • Irregular heart rhythms

      • Muscle Spasms 

      • Dry Mouth 

      • Impotence 

      • Drowsiness 

      • Sleep difficulty 

      • Confusion 

      • Rash 

      • Increasing Pain 

      • Other: _______ 

15. Which parts of your life were the most affected because of your pain?  (Check all that apply)

• Regular work outside the home

• Work around the house

• Relationships with family

• Relationships with others

• Ability to exercise/participate in sports

• Ability to participate in hobbies/recreation

• Finances

• Mood

• Sleep

• Other: ________

16. How long have you been receiving nerve blocks at CPM?

      • Less than 2 months

      • 2-6 months

      • 7-12 months

      • 1-2 years 

      • 3-4 years 

      • Greater than 4 years

17. How frequently do you receive nerve blocks?

      • Twice weekly

      • Weekly

      • Every 2 weeks

      • Every 3 weeks

      • Every 4 weeks 

      • Greater than every 4 weeks

 18. Please indicate how soon you experience relief after receiving nerve blocks.

     • Immediate

     • ________Hours

     • ________Days

19. After a nerve block, how long do you get pain relief before the pain returns to the level it was before the nerve block?

 • Less than 1 day

 • 1-2 days

 • 3-6 days

 • 7-10 days

 • 2-3 weeks

 • If >4 weeks, then how long?   ________

20. Overall, how helpful have you found nerve blocks in reducing your pain? 

(0=not at all to 10=complete pain relief):

               0    1    2    3    4    5    6    7    8    9    10

21. Were you able to stop or reduce the dosage of any medications once you started getting nerve blocks?             

       (Check all that apply)

  • No, I am still on the same medications as I was before the nerve blocks.

  • Yes, I have been able to stop or reduce the dosage of the following medications (check all that apply):

• Topical rubs

• Acetaminophen (Tylenol)

• Codeine (Tylenol #3, Codeine Contin) 

• Oxycodone (Percocet, OxyContin, OxyNEO) 

• Hydromorphone (Dilaudid, Jurnista, Hydromorph Contin) 

• Morphine (MS Contin, MEslon, Kadian) 

• Tramadol (Ultram, Tramacet, Tridural, Durela 

• Tapentadol (Nucynta)  

• Fentanyl patch 

• Buprenorphine (BuTrans patch) 

• Buprenorphine buccal film (Belbuca)

• Tricyclics (Amitriptyline, Nortriptyline) 

• Gabapentin (Neurontin), Pregabalin (Lyrica) 

• Duloxetine (Cymbalta), Venlafaxine (Effexor) 

• Topiramate (Topamax) 

• Nabilone (Cesamet) 

• Other: _____________________________________________

22. Overall, how helpful have you found nerve blocks in improving your function and quality of life?

       (0=not at all helpful; 10=functioning the same as I was prior to developing chronic pain):

Not at all helpful    0    1    2    3    4    5    6    7    8    9    10    Completely restored my previous function

23. Please rate your quality of life before and after starting nerve blocks on the following scales (On a scale of 0-10, with 0 being “unable to do at all” and 10 being “can do all without difficulty”):        

BEFORE BLOCKS                                        AFTER BLOCKS

Family/Home Responsibilities (includes chores and errands performed around the house)

(0  1  2  3  4  5  6  7  8  9  10)                     (0  1  2  3  4  5  6  7  8  9 10)

Recreation (includes hobbies, sports, and other leisure time activities)

(0  1  2  3  4  5  6  7  8  9  10)                    (0  1  2  3  4  5  6  7  8  9  10)

Social Activity   (includes activities with friends like dining out or going to concerts)

(0  1  2  3  4  5  6  7  8  9  10)                     (0  1  2  3  4  5  6  7  8  9  10)

Occupation  (this refers to activities related to your job or daily occupation)

(0  1  2  3  4  5  6  7  8  9  10)                     (0  1  2  3  4  5  6  7  8  9  10)

Sexual Behavior  (this refers to the frequency and quality of your sex life)

(0  1  2  3  4  5  6  7  8  9  10)                     (0  1  2  3  4  5  6  7  8  9  10)

Self Care  (includes activities like taking a shower or getting dressed)

(0  1  2  3  4  5  6  7  8  9  10)                     (0  1  2  3  4  5  6  7  8  9  10)

Life-Support Activities  (this refers to basic activities like eating, sleeping and breathing)

(0  1  2  3  4  5  6  7  8  9  10)                     (0  1  2  3  4  5  6  7  8  9  10)

24. Please rate your average pain scores before and after starting nerve blocks on the following scales (Scales of 0-10, with 0 being “no pain” and 10 being “the worst pain imaginable”): 

                                             BEFORE BLOCKS                                                  AFTER BLOCKS

 Worst pain                           (0  1  2  3  4  5  6  7  8  9  10)               (0  1  2  3  4  5  6  7  8  9  10)

 Least pain                            (0  1  2  3  4  5  6  7  8  9  10)               (0  1  2  3  4  5  6  7  8  9  10)

Average pain                       (0  1  2  3  4  5  6  7  8  9  10)                (0  1  2  3  4  5  6  7  8  9  10)

25. Have you experienced any significant side effects of having nerve blocks?

• No

• Yes - please check all that apply below:

  • Nausea

  • Headaches

  • Constipation 

  • Low Blood Pressure 

  • Irregular Heart Rhythms

  • Muscle Spasms 

  • Dry Mouth 

  • Impotence 

  • Drowsiness 

  • Insomnia 

  • Confusion 

  • Rash 

  • Increasing Pain 

  • Bruising 

  • Swelling 

  • Weakness in a limb (arm or leg) 

  • Passing out 

  • Other: (text box_______) 

26. If you experienced side effects after nerve blocks, were any of these bad enough for you to consider stopping nerve blocks?

 • No

 • Yes

28. Answer this question only if you are currently working:

  If you are working how much did nerve blocks help you to return to or stay at work? 

   (0 = no impact on working;   10 = a huge impact on being able to work)               

  No Impact    0    1    2    3    4    5    6    7    8    9    10   Huge Impact

29. Answer this question only if you are currently working:

    If the Ontario government decided to stop paying for nerve blocks, how would it affect your ability to work?

  • Could not work full time 

  • Could not work at all 

  • No effect - nerve blocks had no influence on being able to work 

30. If the Ontario government decided to stop paying for nerve blocks, how else would your life change? (Check all that apply):

 • Less ability to provide self-care

 • Less ability to perform home chores

 • Less ability to be a good parent/spouse

 • Less ability to enjoy hobbies/recreational activities

 • Less ability to socialize

 • Worse mood

 • Worse sleep

 • Other: ______________________________________________ 

 31. If the Ontario government decided to stop paying for nerve blocks, how else would you treat your pain?  (Check all that apply)

 • More visits to my family doctor

 • More visits to the emergency department

 • More prescription pain medication

 • Seek out street drugs

 • Use cannabis (marijuana) for my pain

 • Seek out surgery for my pain

 • Don’t know what I would do

 • Other: _______________________________________________ 

32. In order to better understand what impact nerve block treatments have had on your pain and quality of life, would you allow us to look into your medical chart at CPM? This would allow us to compare the most recent pain scores and quality of life scores with the scores when you first came to CPM.

 • Yes   (If Yes, please type in your name: _________________________________  )

 • No

33. Please feel free to share any other information on how nerve blocks have affected your pain and quality of life? Please type below.

_______________________________________________ 

Thank you for taking the time to complete this questionnaire. Please return the tablet to the reception staff.

Appendix 2: Representative quotes

**Table 12:** Representative quotes

Theme	Representative quotes
Increased functioning	● “It has kept me mobile and I can walk longer distance”
	● “These blocks allow me to function, without them, I would be bed bound more or less, what is the point?”
	● “I am able to function (even for a short period of time), can complete hygiene with little assistance, enjoy quality time with my family (even for brief moments), depression/suicidal thoughts diminished - ceased trips (there were many) to Emergency room, and being stigmatized.”
	● “I could not get out of a chair before. I could not function. I exercise in a pool now and I walk. Without the needles, I don’t know what will happen to me.”
	● “Without nerve blocks, I wouldn’t be able to work, and I’d probably be dead by now.”
	● “I am able to sit and stand for longer periods of time. I can be in the car and not be in pain constantly. I can do my own grocery shopping and some housework. I don't like the needles but it gives me 3-4 + days where I am not in constant pain. I need nerve blocks to help with the constant pain. Please continue with the nerve blocks.”
	● “Without the nerve blocks, any activity involving standing, walking, sitting etc. would be limited to 3 to 5 minutes at a time. With the nerve blocks, the time increases to 30 minutes to an hour. Amazing improvement.”
	● “They are a necessary to relieve pain, exercise, stretch and physically move my pelvis for walking, even sleeping.”
	● “Those few days of relief after my nerve blocks is the only time I feel like I want to live. It is the time when I can do things that normal people can do such as household chores, grocery shopping, and all the simple things people take for granted. Those few days are the only time I get any sleep.”
	● “I was in bed in such pain I wanted to die all day and night and now I have a part time job.”
Improved mental health	● “Nerve block treatments have given me the ability to function as a normal human being, have constant support, and given me self-confidence back. Before nerve block treatments, I struggled with a combination of self-pity and worthlessness. That has all changed. It took quite a while to find the right places for injections, but now I know that my treatment plan is doing exactly what it is intended to do.”
	● “I was ready to end my life, just had enough of the constant pain, the horrible suffering, and meds messed you up or made you so sick, wasn’t worth it. The shots give a break in a sea of pain, I need them to continue on...”
	● “Due to winter storms, I missed 3 appointments and my pain increased drastically. Nerve blocks have made my life manageable and totally helped with my depression and desire to seek employment. Nerve blocks have helped with all aspects of my life. I believe that these treatments have saved my life and made me optimistic regarding a happier future.”
	● “My moods are better. I have not suffered the debilitating depression I had before I came for the shots. However, since it has become known about the possibility of losing my lifeline of the nerve blocks, I find I am suffering more and more from depressive episodes. I seriously do not want to go back on opioids, but fear I will have little choice if this lifeline is taken away!!!”
	● “For two years, I was getting blocks in my back with some relief. Two weeks ago, my doctor put blocks in my knees. I felt relief right away. My legs had 0 pain. I even started to high knee march in front of the doctor, I was ready to do jumping jacks. I left the office with a glow on my face. When I got home, I surprised my wife and started dancing around the living room. She was in tears to see me with such relief.- enhances mood The next day, I got on my knees for the first time since my back surgery in 2015. For two days, I had a normal human feeling for life and they want to do something. When the block wore off my depression returned tenfold. I lost interest in life once again.- depression I spent 2 days in bed due to my mental health being effected by the unslot of pain once again. I could write 3000-word paper with no problem on why blocks are needed. Without blocks, I will cost the Government just as much money seeking help from other branches of the health care system such as the Ontario Drug Plan.”
	● “The blocks have allowed me to work full time in my field of study, engineering. More importantly, they decreased my pain enough that I was able to get pregnant and have two children. I used to yell out in pain, cry while vomiting on my bathroom floor, hitting my hand or foot to feel sharp pain anywhere to distract from the overall pain. I was afraid I would kill myself one day in the midst of the worst pain. I no longer worry about this. During the worst of the pain now, I can lie in bed quietly, and wait for it to pass with dignity. The blocks have given me a chance at a real life that I am no longer afraid to live. I just want to work and take care of my children with dignity. Please grant me this.”
Role in pain management	● “Just like previously stated, my injections cut my pain in half. That may not seem ideal, but it is significant to me and the injections help gives my body a break from intense pain in my neck, back, arm and hand. They also help lower my blood pressure, improve my mood and overall quality of life. I have no unwanted side effects and DOES help.”
	● “The blocks provide relief I could not get from anything else including medication. I could not do anything without the needles (nerve block). By the 5th day, I am in excruciating pain and unable to do much of anything. The needles give me immediate relief from my migraine and sciatic pain and take my pain levels down. I have a better quality of life with the nerve blocks.”
	● “Prior to nerve block injections, I spent 70% -80% of my time in bed in terrible pain or as result of side effects from the amount of medication I had to take just to be able tolerate the pain levels I had. The nerve blocks are definitely worth getting in my opinion.”
	● “The blocks have allowed me to work full time in my field of study, engineering. More importantly, they decreased my pain enough that I was able to get pregnant and have two children. I used to yell out in pain, cry while vomiting on my bathroom floor, hitting my hand or foot to feel sharp pain anywhere to distract from the overall pain. I was afraid I would kill myself one day in the midst of the worst pain. I no longer worry about this. During the worst of the pain now, I can lie in bed quietly, and wait for it to pass with dignity. The blocks have given me a chance at a real life that I am no longer afraid to live. I just want to work and take care of my children with dignity. Please grant me this.”
	● “If I can’t receive nerve block injections to relieve the pain, I don't know what I would do because I don't know if I could live with the pain.”
Improved quality of life (QoL)	● “Before nerve blocks I couldn't sleep at night because of pain. During the daytime, I was exhausted from no sleep. I couldn't go outside much, do housework at all. Basically, I was bed ridden. Now, I can do so much more. I actually have a more ‘normal’ life.”
	● “Before receiving the blocks, I was severely depressed and tried to take my life as I felt I was a burden on my wife and family. The blocks have helped me to be able to dress myself, and increase my independence. Also, before, I was unable to play with my children or watch them in their sports. Now, I’m able to partake in family events including eating on a regular basis, which helps manage my diabetes and my sleep patterns better. My quality of life has significantly improved and helps me function on a daily basis.... something that someone without chronic pain won’t understand.”
	● “I did have a couple of months where I was unable to have the nerve blocks so I do know how this affected me in my life. I was unable to get a good night’s sleep as my nerve pain is worse at night. I found that I wasn’t going out as much, my mood was worse which I would take out on my family which in turn hurt my relationship with my husband. I think that unless a person is dealing with chronic pain or knows someone with chronic pain they have no idea what it is like to deal with it every day so they should not be making the decision to take away the nerve blocks.”
	● “I feel that I have a chance at life now.”
	● “For 25 years, I thought it was helpless and had no hope I would ever live life without severe pain and disability. Since getting nerve blocks, it gave me hope and allowed me to live with pain that was much more tolerable and my life is now significantly better and improved my quality of life. I believe without pain blocks, my pain would be very severe again… a major setback limiting my everyday life.”
	● “Before nerve blocks, I was unable to function, the pain was crippling, I could not stand nor sit for long periods of time and would be almost paralyzed at some points, I stopped social interactions and pulled away from family as I physically could not attend functions. Since I began nerve blocks my life has resumed. I can work a full shift, I can go out with friends and family, I can participate in physical activities, and I can be me again. if the nerve blocks were taken away I would cease to function as a contributing member of society.”
	● “Nerve blocks gave me my life back. I was able to plan things, accept invitations for Christmas, go to the movies, play golf, and enjoy with my friends. Without nerve blocks, I was just in pain. With nerve blocks, I got my life back and could feel joy again…”
	● “They have effected every aspect of my life. Being able to help support my family and actually be a part of life is amazing!”
Decreased reliance on other pain relief methods	● “If I even miss 1 week, it affects me greatly. I am in constant pain and I would have to increase my opioids. If this happens, more and more people will have to do the same, and that is something I thought the government wanted less people on opioids; not creating another opioid crisis.”
	● “Having the nerve blocks have also helped the psychological dealing of pain and inabilities. It is a relief of being able to reduce pain medication by more than half the original amounts prior to blocks. I wouldn’t want to have my life go in reverse of prior to the blocks. The blocks enabling the not using a cane also eliminated the pain I was having in my shoulder from cane use. Being able to reduce medications of all types also eliminates the concern of future damage to the liver. Nerve blocks are also a good practice to help save lives from people dying of drug overdoses. I also feel that there would be less people shopping around for pain medication to help eliminate pain issues that nerve blocks help with. NERVE BLOCKS SAVE LIVES AND PROVIDE BETTER QUALITY OF LIFE!!!!!”
	● “I would be lost. I have been through a lot. This is my hope for a future. I would have to start looking into surgery options, increase my pain medication (I can’t drive after taking medication), I would be home bound.”
	● “Nerve blocks saved me from a life of opioid addiction and gives me the ability to work and make an income to help support my family.”
	● “These nerve blocker treatments are absolutely essential to my quality of life. I will end up taking a high dose of opioid medication again, which I have put a lot of pain and effort into getting off of; leaving me nearly unable to function again, wasting my life away, in order to not be in severe pain. And I will also be taking up unnecessary space and resources at Emergency rooms. And, I just might entertain the idea of using street drugs to treat my pain, out of pure desperation. These are not acceptable options to me. Chronic pain is enough to drive any human being mad, and straight to suicide, which is why those of us who suffer from chronic pain do anything and everything we can to treat it. These nerve blocker injections are very painful, but we gladly take them because they are very effective. Or we would not do it.”
	● “Nerve blocks have given me safe treatment options instead of using illegal medications. This has impacted my life in many other ways beyond just the treatment. It is a positive safe path to dealing with chronic pain, and without it, my life would be for the worse.”
Concerns regarding proposed defunding of nerve blocks	● “It has given me hope for a fulfilling life after total medical devastation. My life would be over if funding for nerve blocks were cut. I get over 20 injections per visit and at least 8 of those injections are for migraines. The previously proposed cuts would ruin my life and cause me to consider suicide again.”
	● “It’s amazing to me there is a treatment available to help me function daily. I feel very blessed to live in Canada where you have healthcare with people with disability and financial restrictions to help us. People who don’t live with severe chronic pain every single hour of everyday cannot even imagine what we are going through! My question is what would you do if your spouse, your parent, your child, or the person you love was going through this and you had the power to keep the treatment so that person would live a normal life as possible? How would you feel to see your love one suffer every single day knowing that you could have done something about it? HEALTH IS VERY FRAGILE AND PRECIOUS! I believe in a long run it would cost the government more! There are a lot of area with less importance that you could cut!”
	● “I would like to add that reducing this medical treatment is short-sighted. I suspect that visits to medical professionals will increase exponentially, that more medication will be prescribed, and the people afflicted with chronic pain will experience higher suicide levels. The cost for this should exceed present levels of funding. I wonder if the bureaucrats and politicians can sleep with that on their collective conscience.”
	● “Please do not stop the availability of nerve blocks. You have no idea what they do for us unless you were put in the same position. Please don't take away my life.”

## References

[1] Schopflocher D, Taenzer P, Jovey R (2011). The prevalence of chronic pain in Canada. Pain Res Manag.

[2] Reitsma ML, Tranmer JE, Buchanan DM, Vandenkerkhof EG (2011). The prevalence of chronic pain and pain-related interference in the Canadian population from 1994 to 2008. Chronic Dis Inj Can.

[3] Campbell F, Hudspith M, Choiniere M (2021). An Action Plan for Pain in Canada. https://www.canada.ca/content/dam/hc-sc/documents/corporate/about-health-canada/public-engagement/external-advisory-bodies/canadian-pain-task-force/report-2021-rapport/report-rapport-2021-eng.pdf.

[4] Wilson MG, Lavis JN, Ellen ME (2015). Supporting chronic pain management across provincial and territorial health systems in Canada: findings from two stakeholder dialogues. Pain Res Manag.

[5] Ray WA, Chung CP, Murray KT, Hall K, Stein CM (2016). Prescription of long-acting opioids and mortality in patients with chronic noncancer pain. JAMA.

[6] Busse JW, Wang L, Kamaleldin M (2018). Opioids for chronic noncancer pain: a systematic review and meta-analysis. JAMA.

[7] Wilton J, Abdia Y, Chong M (2021). Prescription opioid treatment for non-cancer pain and initiation of injection drug use: large retrospective cohort study. BMJ.

[8] Skelly AC, Chou R, Dettori JR (2020). Noninvasive Nonpharmacological Treatment for Chronic Pain: A Systematic Review Update. https://www.ncbi.nlm.nih.gov/books/NBK556229/.

[9] McDonagh MS, Selph SS, Buckley DI (2020). Noninvasive Nonpharmacological Treatment for Chronic Pain: A Systematic Review Update. https://www.ncbi.nlm.nih.gov/books/NBK556277/.

[10] Chou R, Hartung D, Turner J (2020). Opioid Treatments for Chronic Pain. https://www.ncbi.nlm.nih.gov/books/NBK556253/.

[11] (2023). Ontario must fix excessive use of pain blockers. https://www.thestar.com/opinion/editorials/2020/09/29/ontario-must-fix-excessive-use-of-pain-blockers.html.

[12] Imamura M, Imamura ST, Targino RA (2016). Paraspinous lidocaine injection for chronic nonspecific low back pain: a randomized controlled clinical trial. J Pain.

[13] Jacobs H, Weinberg J, O'Connell J, Buckley N, Nussbaum D, Ko G (2019). Nerve blocks lead to improved quality of life. Pract Pain Manag.

[14] (2023). ‘That’s an injection mill.’ Ontario’s top-billing pain doctors capitalize on province’s lax rules, running up the public’s tab for chronic pain management. https://www.thestar.com/news/investigations/2020/09/28/thats-an-injection-mill-ontarios-top-billing-pain-doctors-capitalize-on-provinces-lax-rules-running-up-the-publics-tab-for-chronic-pain-management.html.

[15] (2023). OHP Standard: Image Guidance When Administering Nerve Blocks for Adult Chronic Pain. https://policyconsult.cpso.on.ca/.

[16] Griffith LE, Cook DJ, Guyatt GH, Charles CA (1999). Comparison of open and closed questionnaire formats in obtaining demographic information from Canadian general internists. J Clin Epidemiol.

[17] Farrar JT, Polomano RC, Berlin JA, Strom BL (2010). A comparison of change in the 0-10 numeric rating scale to a pain relief scale and global medication performance scale in a short-term clinical trial of breakthrough pain intensity. Anesthesiology.

[18] Grant M, O-Beirne-Elliman J, Froud R, Underwood M, Seers K (2019). The work of return to work. Challenges of returning to work when you have chronic pain: a meta-ethnography. BMJ Open.

[19] Svanholm F, Liedberg GM, Löfgren M, Björk M (2022). Factors of importance for return to work, experienced by patients with chronic pain that have completed a multimodal rehabilitation program - a focus group study. Disabil Rehabil.

[20] Deng G, Gofeld M, Reid JN, Welk B, Agur AM, Loh E (2021). A retrospective cohort study of healthcare utilization associated with paravertebral blocks for chronic pain management in Ontario. Can J Pain.

[21] (2023). NICE Guideline [NG59]. Low back pain and sciatica in over 16s: assessment and management. https://www.nice.org.uk/guidance/ng59/chapter/Recommendations.

[22] Manchikanti L, Kaye AD, Soin A (2020). Comprehensive evidence-based guidelines for facet joint interventions in the management of chronic spinal pain: American Society of Interventional Pain Physicians (ASIPP) guidelines facet joint interventions 2020 guidelines. Pain Physician.

[23] Busse JW, Mahmood H, Maqbool B (2015). Characteristics of patients receiving long-term opioid therapy for chronic noncancer pain: a cross-sectional survey of patients attending the Pain Management Centre at Hamilton General Hospital, Hamilton, Ontario. CMAJ Open.

[24] Zou K, Wong J, Abdullah N, Chen X, Smith T, Doherty M, Zhang W (2016). Examination of overall treatment effect and the proportion attributable to contextual effect in osteoarthritis: meta-analysis of randomised controlled trials. Ann Rheum Dis.

[25] Jonas WB, Crawford C, Colloca L (2015). To what extent are surgery and invasive procedures effective beyond a placebo response? A systematic review with meta-analysis of randomised, sham controlled trials. BMJ Open.

